# Delusional disorders with religious content

**DOI:** 10.1192/j.eurpsy.2021.2037

**Published:** 2021-08-13

**Authors:** O. Borisova, G. Kopeyko, E. Gedevani, V. Kaleda

**Affiliations:** 1 Investigation Group Of Specific Psychopathological Forms At Department Of Youth Psychiatry, Federal State Budgetary Scientific Institution «Mental Health Research Center», Moscow, Russian Federation; 2 Department Of Youth Psychiatry, FSBSI «Mental Health Research Centre», Moscow, Russian Federation

**Keywords:** schizophrénia, religious delusions, destructive behavior, psychopathology

## Abstract

**Introduction:**

Delusional Disorders with Religious Content (DDRC) require careful study concerning their prevalence, psychopathological heterogeneity and the risk of destructive behavior.

**Objectives:**

To classify the clinical forms of DDRC

**Methods:**

By clinical-psychopathological, follow-up and statistical approaches 2523 cases of patients with mental disorders who received inpatient care in a state clinic for year were analyzed; in 225 cases of total 2523 delusional disorders in schizophrenia (ICD-10: F20.0, F20.01, F20.02) were diagnosed.

**Results:**

The comparative analysis of delusional disorders (225 cases, 100%) with religious (70 cases -31.1%) and non-religious content (155 cases - 69.9%) revealed prevalence of DDRC in non-believers (p <0.01). Delusional destructive behavior occurred in 47.1% of 70 cases in patients with DDRC (15% of total 225).

**Table:** 

Delusional disorders 225 cases (100%)				
	DDRC (70 cases, 31,1%)		Delusional disorders with non-religious content (155 cases - 69.9%)	
	Believers	Non-believers	Believers	Non-believers
Total Cases	18 (8%)	52 (23,1%)	4 (1,8%)	151 (67,1%)
With Destructive behavior	10 (4,4%)	23 (10,2%)	0	61 (27,1%)
	33 (14,6 %)		61 (27,1%)

The predominant content of DDRC (among the Delusions of Possession, Sinfulness/guilt, Messianism, Manichaean and the End-world Delusions) was the Delusions of Possession - 36.8%. Psychopathological heterogeneity of DDRC was identified and specific types of DDRC were described.

**Conclusions:**

DDRC is associated with the development of massive psychopathological symptoms and significant severity, and often accompanied by various forms of destractive behavior. This circumstance requires constant and careful management of these patients, collection of their religious history and asks for specific therapeutic approaches.

**Disclosure:**

No significant relationships.

